# Performance evaluation of automated white matter hyperintensity segmentation algorithms in a multicenter cohort on cognitive impairment and dementia

**DOI:** 10.3389/fpsyt.2022.1010273

**Published:** 2023-01-12

**Authors:** Malo Gaubert, Andrea Dell’Orco, Catharina Lange, Antoine Garnier-Crussard, Isabella Zimmermann, Martin Dyrba, Marco Duering, Gabriel Ziegler, Oliver Peters, Lukas Preis, Josef Priller, Eike Jakob Spruth, Anja Schneider, Klaus Fliessbach, Jens Wiltfang, Björn H. Schott, Franziska Maier, Wenzel Glanz, Katharina Buerger, Daniel Janowitz, Robert Perneczky, Boris-Stephan Rauchmann, Stefan Teipel, Ingo Kilimann, Christoph Laske, Matthias H. Munk, Annika Spottke, Nina Roy, Laura Dobisch, Michael Ewers, Peter Dechent, John Dylan Haynes, Klaus Scheffler, Emrah Düzel, Frank Jessen, Miranka Wirth, for the DELCODE study group, Amthauer Holger

**Affiliations:** ^1^German Center for Neurodegenerative Diseases, Dresden, Germany; ^2^Department of Neuroradiology, Rennes University Hospital (CHU), Rennes, France; ^3^Department of Neuroradiology, Charité – Universitätsmedizin Berlin, Corporate Member of Freie Universität Berlin and Humboldt-Universität zu Berlin, Berlin, Germany; ^4^Department of Nuclear Medicine, Charité – Universitätsmedizin Berlin, Corporate Member of Freie Universität Berlin and Humboldt-Universität zu Berlin, Berlin, Germany; ^5^Clinical and Research Memory Center of Lyon, Lyon Institute for Elderly, Hospices Civils de Lyon, Lyon, France; ^6^Normandie Univ, UNICAEN, INSERM, U1237, PhIND “Physiopathology and Imaging of Neurological Disorders,” Institut Blood and Brain @ Caen-Normandie, Caen, France; ^7^Neuroscience Research Centre of Lyon, INSERM 1048, CNRS 5292, Lyon, France; ^8^German Center for Neurodegenerative Diseases, Rostock, Germany; ^9^Department of Biomedical Engineering, Medical Image Analysis Center (MIAC) and qbig, University of Basel, Basel, Switzerland; ^10^German Center for Neurodegenerative Diseases, Magdeburg, Germany; ^11^German Center for Neurodegenerative Diseases, Berlin, Germany; ^12^Department of Psychiatry, Charité – Universitätsmedizin Berlin, Berlin, Germany; ^13^Department of Psychiatry and Psychotherapy, Charité - Universitätsmedizin Berlin, Berlin, Germany; ^14^Centre for Clinical Brain Sciences, University of Edinburgh and UK Dementia Research Institute (DRI), Edinburgh, United Kingdom; ^15^Department of Psychiatry and Psychotherapy, School of Medicine, Technical University of Munich, Munich, Germany; ^16^German Center for Neurodegenerative Diseases, Bonn, Germany; ^17^Department of Neurodegenerative Disease and Geriatric Psychiatry, University of Bonn Medical Center, Bonn, Germany; ^18^German Center for Neurodegenerative Diseases, Göttingen, Germany; ^19^Department of Psychiatry and Psychotherapy, University Medical Center Göttingen, University of Göttingen, Göttingen, Germany; ^20^Department of Medical Sciences, Neurosciences and Signaling Group, Institute of Biomedicine (iBiMED), University of Aveiro, Aveiro, Portugal; ^21^Leibniz Institute for Neurobiology, Magdeburg, Germany; ^22^Department of Psychiatry, Medical Faculty, University of Cologne, Cologne, Germany; ^23^German Center for Neurodegenerative Diseases, Munich, Germany; ^24^Department of Psychiatry and Psychotherapy, University Hospital, Ludwig Maximilian University of Munich (LMU), Munich, Germany; ^25^Munich Cluster for Systems Neurology (SyNergy) Munich, Munich, Germany; ^26^Ageing Epidemiology Research Unit (AGE), School of Public Health, Imperial College London, London, United Kingdom; ^27^Sheffield Institute for Translational Neuroscience (SITraN), University of Sheffield, Sheffield, United Kingdom; ^28^Department of Psychosomatic Medicine, Rostock University Medical Center, Rostock, Germany; ^29^German Center for Neurodegenerative Diseases, Tübingen, Germany; ^30^Department of Psychiatry and Psychotherapy, University of Tübingen, Tübingen, Germany; ^31^Department of Neurology, University of Bonn, Bonn, Germany; ^32^MR-Research in Neurosciences, Department of Cognitive Neurology, Georg-August-University of Göttingen, Göttingen, Germany; ^33^Bernstein Center for Computational Neuroscience, Charité – Universitätsmedizin, Berlin, Germany; ^34^Department for Biomedical Magnetic Resonance, University of Tübingen, Tübingen, Germany; ^35^Institute of Cognitive Neurology and Dementia Research (IKND), Otto-von-Guericke University, Magdeburg, Germany; ^36^Excellence Cluster on Cellular Stress Responses in Aging-Associated Diseases (CECAD), University of Cologne, Köln, Germany

**Keywords:** white matter hyperintensities segmentation, evaluation, FLAIR, deep learning, aging, Alzheimer’s disease

## Abstract

**Background:**

White matter hyperintensities (WMH), a biomarker of small vessel disease, are often found in Alzheimer’s disease (AD) and their advanced detection and quantification can be beneficial for research and clinical applications. To investigate WMH in large-scale multicenter studies on cognitive impairment and AD, appropriate automated WMH segmentation algorithms are required. This study aimed to compare the performance of segmentation tools and provide information on their application in multicenter research.

**Methods:**

We used a pseudo-randomly selected dataset (*n* = 50) from the DZNE-multicenter observational Longitudinal Cognitive Impairment and Dementia Study (DELCODE) that included 3D fluid-attenuated inversion recovery (FLAIR) images from participants across the cognitive continuum. Performances of top-rated algorithms for automated WMH segmentation [Brain Intensity Abnormality Classification Algorithm (BIANCA), lesion segmentation toolbox (LST), lesion growth algorithm (LGA), LST lesion prediction algorithm (LPA), pgs, and sysu_media] were compared to manual reference segmentation (RS).

**Results:**

Across tools, segmentation performance was moderate for global WMH volume and number of detected lesions. After retraining on a DELCODE subset, the deep learning algorithm sysu_media showed the highest performances with an average Dice’s coefficient of 0.702 (±0.109 SD) for volume and a mean F1-score of 0.642 (±0.109 SD) for the number of lesions. The intra-class correlation was excellent for all algorithms (>0.9) but BIANCA (0.835). Performance improved with high WMH burden and varied across brain regions.

**Conclusion:**

To conclude, the deep learning algorithm, when retrained, performed well in the multicenter context. Nevertheless, the performance was close to traditional methods. We provide methodological recommendations for future studies using automated WMH segmentation to quantify and assess WMH along the continuum of cognitive impairment and AD dementia.

## Introduction

White matter hyperintensities (WMH) of presumed vascular origin are defined as bright foci in cerebral white matter (WM) on magnetic resonance imaging (MRI) T2-weighted or fluid-attenuated inversion recovery (FLAIR) images (1). WMH are found prominently in older adults and in patients diagnosed with cerebral small vessel disease and neurodegenerative diseases, such as Alzheimer’s disease (AD) (1, 2). Generally associated with covert neurological, cognitive and physical conditions (1), WMH appear to play an important role in AD (3, 4). Manual segmentation is currently considered the best method to quantify these abnormalities. However, this procedure is a time-consuming task prone to errors (5).

To overcome these drawbacks, many tools have been developed to automatically detect and segment WMH [see Supplementary Information from Vanderbecq et al. for a recent review (6)]. The algorithms applied by these tools include Markov random fields (7), support vector machines (8) or K-nearest neighbors (9). Recently, a new class of algorithms has emerged based on deep neural networks, yielding top-ranking results in the recent Medical Image Computing and Computer Assisted Intervention (MICCAI) challenge on WMH segmentation (10). Nevertheless, the choice of a relevant method for WMH detection is difficult with a variety of available tools and evaluation criteria. Moreover, most of the original studies tested the methods on images acquired from a single MRI scanner, such that robustness and reliability still need to be proven for multicenter data.

Regular challenges, as provided by the MICCAI society, constitute an excellent competition for state-of-the-art segmentation techniques on well-documented datasets. For example, the last MICCAI challenge on WMH segmentation (10) used a manually segmented dataset in a multicenter context. Unfortunately, the WMH segmentation tools most frequently used in research studies are often neglected in the MICCAI challenge, thus limiting our understanding of the evolution and performance of new tools compared to already published ones. Recent studies compared multiple automatic WMH segmentation tools using multicenter databases (6, 11, 12). With a rigorous methodology, Heinen et al. (11) showed best performances for k-nearest neighbor classification with tissue type priors (kNN-TMP) (13) and, to a lesser extent, for lesion growth algorithm (LGA) (7) and lesion prediction algorithm (LPA) (14), both included in lesion segmentation toolbox (LST). Vanderbecq et al. (6) showed that a new algorithm using deep learning performed well on a research dataset, especially when retrained, and that more traditional algorithms worked best on a clinical dataset. Finally, Khademi et al. (12) observed better performances for new algorithms compared to partial volume average modeling or LST LPA algorithms. In general, it thus appears that algorithms using deep learning methods may outperform more traditional tools. Notably though, existing studies have often used the default parameters for all tools, which can obscure the real potential of the segmentation methods (6, 11). Moreover, the description of the manual WMH segmentation is often scarce, without reference to guidelines, thus impeding accessibility and reproducibility of findings (15).

The objective of the present study was to provide evidence on broadly applicable tools to automatically segment WMH in a multicenter context on cognitive impairment and dementia. To this end, we relied on 50 manual reference WMH segmentations of FLAIR images from the German multicenter observational DZNE-Longitudinal Cognitive Impairment and Dementia Study [DELCODE (16)]. We assessed and compared five automated segmentation tools: three are extensively used in the literature, namely the Brain Intensity Abnormality Classification Algorithm (BIANCA) (9), LST LGA (7) and LST LPA (14), while the two others are based on neural networks, namely pgs (17) and sysu_media (18). The latter are the two highest ranked tools in the MICCAI challenge on WMH segmentations at the time of this study. Moreover, to compare the impact of training on segmentation performance, sysu_media was assessed with two training configurations: the default, originating from MICCAI challenge and a tuned model obtained from retraining the neural network parameters using an independent subset comprising 20 manual reference segmentations (RS) based on the DELCODE study. Evaluation of performances of the WMH segmentation algorithms was based on objective measures related to the three measures of interest to describe WMH according to the standards for reporting vascular changes in neuroimaging (STRIVE): volume, number of lesions and their location (1).

## Materials and methods

### Study sample

Fifty older adults (≥60 years) were pseudo-randomly selected from the cohort of the German multicenter longitudinal observational DELCODE study. The detailed protocol of this study is described in a previous report (16). DELCODE was registered at the German Clinical Trials Register DRKS00007966 (2015/05/04) and approved by the local ethics committees of all participating institutions in accordance with the Declaration of Helsinki. The DELCODE study was approved under a harmonized vote, in which the approval process is coordinated by the ethical committee of the medical faculty of the University of Bonn (Ethik-Kommission der Medizinische Fakultät, Rheinische Friedrich-Wilhelms-Universität, Bonn, Germany; Registration number: 117/13) in charge. Any change of the protocol and the consents have to be approved by all ethic committees. The participants were selected from different diagnostic groups across the cognitive continuum of Alzheimer’s disease meeting the diagnostic criteria for cognitive healthy (HC), subjective cognitive decline (SCD), mild cognitive impartment (MCI) or AD dementia. Demographic information is summarized in Table 1. Briefly, the average age was 71.16 years [range: (61.13–80.9)], the male/female ratio was 21/29, the average years of education was 13.6 [range: (8–20)] and the number of HC, SCD, MCI, and AD participants were 12, 13, 13, and 12, respectively. For this study, participants from each diagnostic group were matched by age, sex, level of education, and assessment site. All participants received detailed study instructions and gave written informed consent prior to study participation.

**TABLE 1:** Demographic characteristics of the selected DELCODE dataset used in this study.

Sample characteristics	*N* = 50
Age	71.16 (5.80) [61.13 80.90]
**Sex**	
Female	29 (58%)
Male	21 (42%)
Years of education	13.60 (3.14) [8 20]
**Group**	
HC	12 (24%)
SCD	13 (26%)
MCI	13 (26%)
AD	12 (24%)

Mean (SD) [range] for numerical variables. Frequency (%) for categorical variables. HC, healthy control; SCD, subjective cognitive decline; MCI, mild cognitive impairment; AD, Alzheimer’s Disease; SD, standard deviation.

Since two segmentation tools required a training dataset (BIANCA and sysu_media), the dataset of 50 participants were split into two subsets: 20 subjects were randomly selected as training subset, while the other 30 subjects were used for the performance evaluation of the tools solely. For both subsets, the numbers of subjects from each site were equally distributed.

### MRI acquisition

All participants underwent an MRI acquisition on a 3 Tesla Siemens Healthineers scanner (Erlangen, Germany) in one of the 10 recruitment sites associated to the DELCODE study. Scanners comprised 3 Magnetom Trio TIM systems, 2 Magnetom Verio systems, 1 Magnetom Prisma system, and 4 Magnetom Skyra systems. As described previously, the MRI protocols were standardized across all sites (16). The acquisition protocol included a T1-weighted (T1w) 3D magnetization prepared rapid gradient echo imaging (MPRAGE) and a 3D FLAIR scans. For the 3D T1w sequence, the acquisition parameters were the following: repetition time (TR) = 2.5 s, echo time (TE) = 4.33 ms, flip angle = 7°, resolution = 1 × 1 × 1 mm^3^, matrix = 256 × 256, 192 slices. For the 3D FLAIR sequence, the acquisition parameters were as follows: TR = 5 s, TE = 394 ms, inversion time = 1.8 s, resolution = 1 × 1 × 1 mm^3^, matrix = 256 × 256, 192 slices.

### Reference segmentation procedure

Manual segmentations were performed on FLAIR images of the 50 participants (thereafter named RS) by one trained rater (I.Z.) after a phase of iterative pre-training on a set of pre-selected images under the supervision of methodological and/or clinical experts (M.W., C.L., and A.G.-C.) following existing neuroimaging standards (1). The rater was blinded to the diagnosis status of participants and study site. The segmentation was performed using ITK-SNAP version 3.6.0^1^ (19) on raw FLAIR and T1w images. The step-by-step manual WMH segmentation protocol was carefully documented to ensure reproducibility and is available from the corresponding authors on reasonable request. Briefly, after checking the quality of the FLAIR and T1w images, the image contrast was adjusted based on one clearly visible WMH. Then, paintbrush mode (or polygon mode for larger clusters) was used to manually delineate each voxel corresponding to WMH. For large WMH areas, the “Active contour segmentation” mode was used to automatically delineate the contours of the lesion before manually checking carefully the contours of the lesions in all slices. Of note, lesions were checked in all orientations (axial, sagittal, and coronal). In accordance to STRIVE (1), the following general rules were applied: WM regions were segmented only, using additionally the T1w scans for more precision on cerebral compartments when needed; cerebrum was segmented only, excluding brainstem, cerebellum and non-brain tissues such as meninges; choroid plexus was excluded as well; interventricular lesions were segmented only on the level of the corpus callosum, but not in the septum.

### Automated WMH segmentation methods

Five freely available algorithms have been used to automatically segment WMH on either individual FLAIR or both T1w and FLAIR images. Three algorithms mostly used in the literature were tested: BIANCA (9), part of FMRIB software library (FSL) toolbox, and LGA (7) and LPA (14), both part of LST, an extension for Statistical Parametric Mapping (SPM).^2^ The two other tools were the best rated algorithms of the 2017 MICCAI challenge on WMH segmentation challenge at the date of today^3^ : pgs algorithm which used UNet with highlighted foreground (17) and sysu_media algorithm based on two-channel U-Net (18).

The BIANCA algorithm is directly implemented in FSL version 6.0.4. The input of BIANCA was a FLAIR image only. Brains were extracted and a light bias correction was applied by using FSL BET (20) (with option –f .2 and –B). For the LST, we used the version 2.0.15 with SPM12 release 7,487 running on Matlab R2018a (MathWorks, Natick, USA). LST LGA requires a T1w and FLAIR images, the latter being automatically registered to the corresponding T1w image by LST. LST LPA supports two modalities and both variations were tested: either FLAIR only, or T1w and FLAIR images as inputs. Again, for the latter, a registration from FLAIR space to T1w space is automatically performed by the toolbox. No other preprocessing was included for LST LGA or LPA. pgs and sysu_media toolboxes can be found online on Docker Hub under the name *wmhchallenge/pgs* and *wmhchallenge/sysu_media*, respectively. For pgs algorithm, a T1w and a FLAIR images are required. Thus, a registration with individual FLAIR images as reference and T1w images as image to move were performed using SPM “Estimate and Reslice” module with default parameters prior to the use of pgs algorithm. No other preprocessing was included. The model weights used for pgs were the same as used during the MICCAI challenge [see Kuijf et al. (10) for more details]. For sysu_media, only FLAIR image were mandatory as an input. sysu_media also required a training dataset. The algorithm was thus tested with two variations: either with the default training dataset from MICCAI challenge as for pgs, or with the training subset based on DELCODE data (see above). For the first one, no additional preprocessing was performed. To retrain the sysu_media UNet, all brain images from both DELCODE training and test subsets have been brain-extracted and lightly bias corrected using FSL BET (20) (option –f .2 and –B) and, then noise corrected using the spatial adaptive non-local means filtering (21) implemented in the Computational Anatomy Toolbox (CAT12.6, version 1450).^4^ For the training process, the same pre- and post-processing and ensemble model as in the original paper was used, but no data augmentation. The same UNet is trained shuffling the training data at the beginning of every epoch. Each of the three ensemble models is saved after 100 epochs or for loss value < 0.30. By loss-function > 0.99, the training of the model is restarted. Other hyperparameters were a batch size of 30 and a learning rate of 0.0002. An implementation of the sysu_media algorithm based on the Python framework Nipype (22) was developed for the analysis with the DELCODE-retrained UNet. This version is available together with the DELCODE weights as an open-source package on GitHub (23).^5^

A summary of the variations and final parameters used for all algorithms can be found in Table 2. Moreover, the evaluations of the best parameter sets are displayed in Supplementary material.

**TABLE 2:** Description of the tools used and their main parameters.

Name	Modalities used	Commentaries	Hyper-parameters	PM threshold
BIANCA	FLAIR	Preprocessing included except for WM mask computing	Lesion/non-lesion points: 2.000/10.000; WM mask threshold: 0.30	0.60
LST LGA	T1w, FLAIR	Preprocessing included	κ = 0.10	0.35
LST LPA	FLAIR	Preprocessing included	None	0.10
LST LPA	T1w, FLAIR	Preprocessing included	None	0.15
pgs	T1w, FLAIR	Training dataset from MICCAI challenge coregistration T1w to FLAIR	None	0.15
sysu_media (default)	FLAIR	Training dataset from MICCAI challenge	None	0.65
sysu_media (retrained)	FLAIR	Preprocessing: brain extraction, SANLM training dataset with 20 participants from DELCODE	None	0.45

PM, probability map; WM, white matter; T1w, T1-weighted image; FLAIR, fluid-attenuated inversion recovery image; SANLM, spatially adaptive non-local means noise correction; MICCAI, the medical image computing and computer assisted intervention; LST LGA/-LPA, lesion segmentation toolbox for SPM using lesion growth algorithm/lesion prediction algorithms; BIANCA, brain intensity abnormality classification algorithm.

### Determination of parameters

Two types of parameters were evaluated to improve WMH segmentation in the DELCODE training subset. The default options available for each tool represented the first type of tuned parameters (hyperparameters). For BIANCA, as described in the online user guide, a WM mask was used to remove non-WM voxels from the segmentation results. The individual WM masks were computed using T1w images and registered to FLAIR space using FSL Flirt with 12 degrees of freedom. The output of the affine registration was a tissue probability map with decimal values ranging from 0 (background) to 1 (WM). The thresholding of these maps was thus tested with values ranging from 0.05 to 0.80 with a step of 0.05. For LST LGA, a threshold for the initial lesion map is required (named kappa). Following the recommendations of the software guideline, we tested values ranging from 0.10 to 0.80 with a step of 0.05.

The second set of parameters tested is related to thresholding the WMH probability map output of the tools. We thus tested values ranging from 0 to 0.95 with a step of 0.05 on each iteration. The final best parameter sets for each tool are reported in Table 2.

Of note, all the output maps from most tools as well as the manual segmentation were in FLAIR space. For the LST LGA and LST LPA with T1w + FLAIR input, the output probability maps were projected back from T1w space to FLAIR space prior to WMH thresholding using the SPM module “Estimate and Reslice” with default parameters except for the interpolation set to trilinear. Finally, clusters with less than 10 contiguous voxels (3D-connectivity of 26 voxels) were removed from all binary maps.

### Performance measures and statistical analysis

After computing the final binary masks of WMH in the DELCODE test subset, the performance of each algorithm was evaluated using different metrics. For volume-based measures, the Sørensen-Dice similarity coefficient (DSC), sensitivity and precision was calculated. With RS_vol_ the volume of WMH load in the RS, AS_vol_ the volume of WMH load of the tested automatic segmentation (AS), and TP_vol_ the volume of the intersection between RS_vol_ and AS_vol_ (also called true positives or hits), the following formulas were applied:

– Sørensen- DSC:


$$\frac{2\times T\,P_{v\,o\,l}}{R\,S_{v\,o\,l}+A\,S_{v\,o\,l}}$$

– Sensitivity:


$$\frac{T\,P_{v\,o\,l}}{R\,S_{v\,o\,l}}$$

– Precision:


$$\frac{T\,P_{v\,o\,l}}{A\,S_{v\,o\,l}}$$

In accordance to these measures, a high sensitivity associated to a lower precision reflects an over-segmentation, while a high precision associated to a lower sensitivity reflects an under-segmentation. The DSC is a summary of these two indexes into a global similarity descriptor. The range of all these indices is comprised between 0 for poor performance (no overlap between RS and AS) and 1 for excellent performance (complete overlap of RS and AS).

The ICC between RS_vol_ and AS_vol_ was also calculated based on two-way random effects, absolute agreement, single rater or ICC (2, 1) as defined in Shrout and Fleiss (24) and Koo and Li (25). All these indexes were computed in the whole brain. Since the location of the WMH lesions is also a measure of interest for WMH, DSC, sensitivity and precision were also computed in 6 regions of interest (ROI), namely the frontal, insular, occipital, parietal and temporal cortices and the corpus callosum based on Hammersmith atlas (26). A complete description of these regions can be found in the Supplementary material.

Besides lesion location and volume, another measure of interest accordingly to STRIVE (1) is the number of WM lesions in the brain. Thus, an algorithm inspired by Commowick et al. (5) was developed to compute the number of lesions correctly detected (TP_lesion_): a true detection in AS was found if any lesion in AS was overlapping the lesion in RS in at least 10% of the number of voxels of the lesion in RS and if this overlap was covering a maximum of 70% of the voxels of the lesion in AS. As for the volume-based performance evaluation, three indices were computed:

– Lesion F1-score (DSC-like score):


$$\frac{2\times T\,P_{l\,e\,s\,i\,o\,n}}{R\,S_{l\,e\,s\,i\,o\,n}+A\,S_{l\,e\,s\,i\,o\,n}}$$

– Lesion sensitivity:


$$\frac{T\,P_{l\,e\,s\,i\,o\,n}}{R\,S_{l\,e\,s\,i\,o\,n}}$$

– Lesion precision:


$$\frac{T\,P_{l\,e\,s\,i\,o\,n}}{A\,S_{l\,e\,s\,i\,o\,n}}$$

where RS_lesion_ and AS_lesion_ are the numbers of lesions found in the reference and AS, respectively.

Finally, to compare the impact of high WMH burden vs. low WMH burden on the accuracy of segmentation, the test subset was split into two equally large subgroups based on global WMH volumes in the RS. A first subgroup of low WMH load included the 10 subjects with the lower global WMH load [mean of 1.65 mL, 0.86 SD, range (0.63–3.30)]. And a second subgroup of high WMH load included the 10 subjects with more extensive global WMH load [mean of 20.22 mL, 14.09 SD, range (8.70–52.68)].

All measures (DSC, sensitivity, precision, and ICC) were categorized into excellent for values above 0.90, good for values between 0.75 and 0.90, moderate for values between 0.50 and 0.75 and poor for values < 0.50 following the classification for ICC proposed by Koo and Li (25).

## Results

### Volume-based results

**FIGURE 1:**
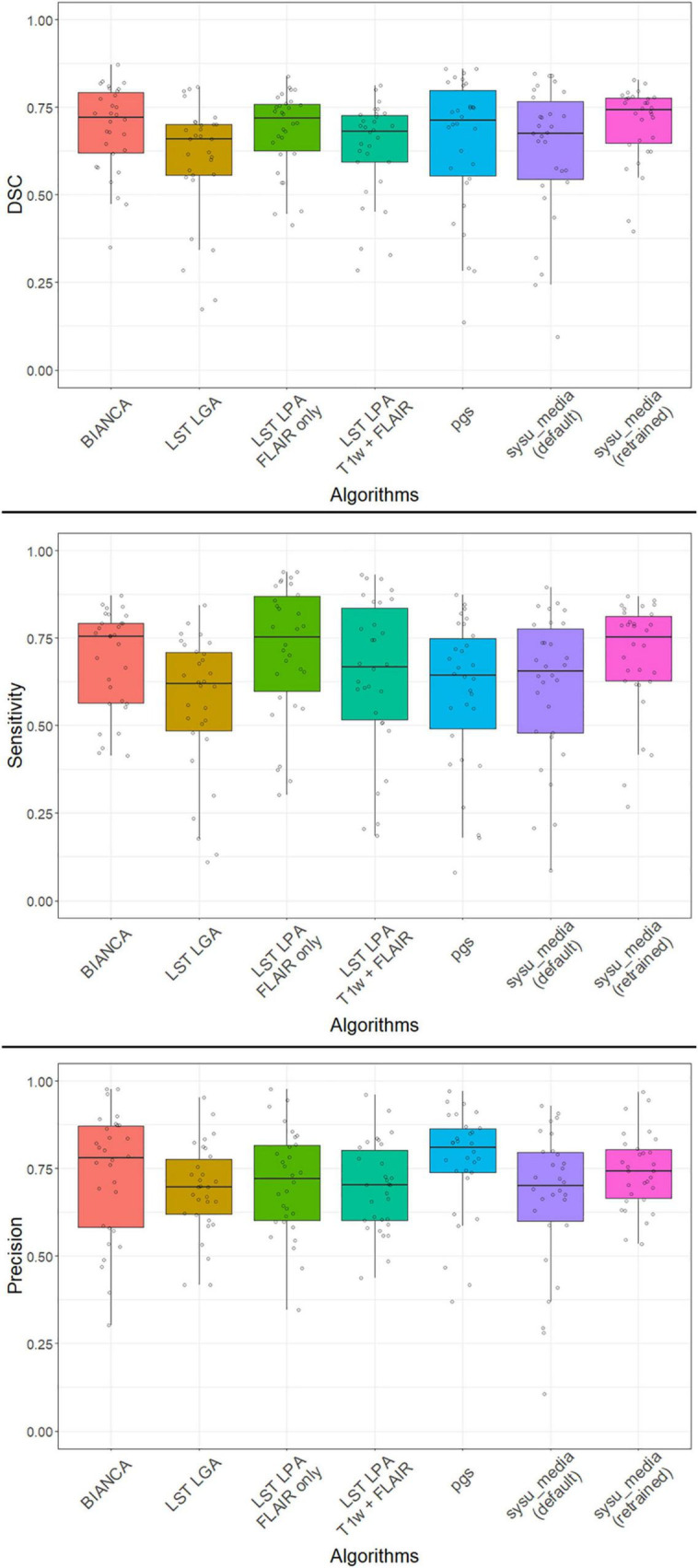
Distribution of performances of all algorithms with DSC **(top)**, sensitivity **(middle)**, and precision **(bottom)** at volume level. The median, the 25–75 percentiles and all individual values are represented. DSC, Sørensen-Dice similarity coefficient; T1w, T1-weighted image; FLAIR, fluid-attenuated inversion recovery image; BIANCA, brain intensity abnormality classification algorithm; LST LGA/-LPA, lesion segmentation toolbox for SPM using lesion growth algorithm/lesion prediction algorithms.

An overview of the results is provided in Table 3 and illustrated in Figure 1. The average similarity coefficient (DSC) was moderate for all segmentation tools with values ranging from 0.602 (± 0.169 SD) for LST LGA to 0.702 (± 0.109) for sysu_media retrained on the DELCODE subset. The sensitivity was moderate with LST LPA performing the best with 0.714 (± 0.190) followed by sysu_media retrained on the DELCODE subset, while LST LGA had the lowest performance with 0.562 (± 0.200). The precision was moderate to good with the best performances for pgs with 0.768 (± 0.151) closely followed by sysu_media retrained on the DELCODE subset with 0.741 (± 0.190) and BIANCA with 0.731 (± 0.179) with the lowest performance measured for sysu_media trained on the MICCAI dataset with 0.665 (± 0.202). Of note, for all these metrics, the variability was consistently the lowest for sysu_media retrained on the DELCODE subset. The resemblance between the RS and each AS characterized by the ICC was good for BIANCA (mean ICC: 0.835) and excellent for all other methods (all mean values > 0.9).

**TABLE 3:** Performances of all algorithms compared to the reference segmentation at volume- and lesion- level.

	Whole brain volumes					Lesions			
**Method**	**Global WMH volume (ml)**	**DSC**	**Sensitivity**	**Precision**	**ICC**	**Number**	**F1-score**	**Sensitivity**	**Precision**
Reference	8.9 ± 11.4	NA	NA	NA	NA	45.2 ± 31.6	NA	NA	NA
BIANCA	6.9 ± 6.7	0.691 ± 0.124	0.687 ± 0.144	0.731 ± 0.179	0.835	38.8 ± 21.4	0.578 ± 0.119	0.573 ± 0.133	0.619 ± 0.166
LST LGA	7.4 ± 9.6	0.602 ± 0.169	0.562 ± 0.200	0.690 ± 0.129	0.943	24.5 ± 15.9	0.466 ± 0.113	0.372 ± 0.112	0.671 ± 0.164
LST LPA FLAIR only	9.5 ± 11.7	0.683 ± 0.114	**0.714 ± 0.190**	0.709 ± 0.150	0.925	32.4 ± 22.2	0.543 ± 0.143	0.490 ± 0.163	0.681 ± 0.199
LST LPA T1w + FLAIR	9.0 ± 11.1	0.632 ± 0.138	0.642 ± 0.222	0.696 ± 0.127	0.907	27.1 ± 19.5	0.536 ± 0.167	0.450 ± 0.181	0.753 ± 0.198
pgs	7.3 ± 9.6	0.651 ± 0.192	0.594 ± 0.214	0.768 ± 0.151	0.959	42.5 ± 30.4	0.635 ± 0.162	0.624 ± 0.182	0.669 ± 0.166
sysu media (default)	8.2 ± 10.5	0.626 ± 0.193	0.610 ± 0.210	0.665 ± 0.202	**0.981**	50.7 ± 28.3	0.569 ± 0.151	**0.631 ± 0.187**	0.536 ± 0.165
sysu media (retrained)	7.7 ± 8.1	**0.702 ± 0.109**	0.695 ± 0.160	**0.741 ± 0.109**	0.914	34.4 ± 25.0	**0.642 ± 0.109**	0.576 ± 0.136	**0.761 ± 0.145**

Average and standard deviation are displayed. mL, milliliter; DSC, Sørensen-Dice similarity coefficient; ICC, intra-class correlation; T1w, T1-weighted image; FLAIR, fluid-attenuated inversion recovery image; LST LGA/-LPA, lesion segmentation toolbox for SPM using lesion growth algorithm/lesion prediction algorithm; BIANCA, brain intensity abnormality classification algorithm; NA, not applicable. Best performance for each evaluation measaures are displayed in bold.

### Number of lesions

The average global score (F1-score) was moderate with best performances for sysu_media retrained on the DELCODE subset with a mean F1-score of 0.642 (± 0.109 SD) closely followed by pgs with 0.635 (± 0.162) and the lowest performance for LST LGA with 0.466 (± 0.113). The average sensitivity to detect lesions of all methods was poor to moderate, with value ranging from 0.372 (± 0.112) for LST LGA to 0.631 (± 0.187) for sysu_media trained on the MICCAI dataset, closely followed by pgs with 0.624 (± 0.182). The average precision was generally good or moderate for all methods with best performance for sysu_media trained on the DELCODE subset with 0.761 (± 0.145) closely followed by LST LPA with FLAIR only and 0.753 (± 0.198) and the lowest performance measured for sysu_media trained on the MICCAI dataset with 0.536 (± 0.165). Again, the variability of all metrics was the lowest for sysu_media retrained on the DELCODE subset, but for sensitivity for which LST LGA had the lowest value. All results presented in this section are displayed in Table 3.

### Performances in ROIs

When looking at the average DSC, sensitivity and precision in the 6 ROIs, we observed that performances of LST LGA, LST LPA and sysu_media retrained on the DELCODE subset were generally better in the corpus callosum compared to the same measures in the other ROIs or at the whole brain level. Specifically, these measures reached above or close to 0.8 for LST LPA with FLAIR and sysu_media retrained on the DELCODE subset. Inversely, these algorithms had the poorest performances in the insular cortex and to a lesser extent in the parietal and temporal cortices compared to the same measures at the whole brain level or in the other ROIs, especially for LST LGA having a DSC and sensitivity below or around 0.5 in these regions. BIANCA had performances similar to the whole brain measures in all ROIs (performances above 0.65), except for the insular and temporal cortices. The sysu_media trained on the MICCAI dataset and pgs had poorest global performances in the corpus callosum compared to the same measures in the other ROIs or at the whole brain level, with sensitivity and DSC values below 0.5, but good precision. Inversely, these algorithms had best performances in the insular cortex with all measures above 0.65. All results presented in this section are summarized in Supplementary material.

### High vs. low global WMH burden

At volume level, the average DSC, sensitivities and precisions were generally higher in the high WMH load group than in the low WMH load group for all methods, with a difference of at least 0.1 reaching more than 0.3 for all indices in sysu_media trained on the MICCAI dataset. When looking at the level of lesions, the observations were more heterogeneous. Except for pgs and sysu_media trained on the MICCAI dataset, where we observed a notable increase in DSC, sensitivity and precision in the high WMH load group compared to the low WMH load group, all other algorithms showed either similar results between the two subgroups or a decrease of all performance metrics in the high WMH load group vs. the low WMH load group. All results presented in this section are displayed in Table 4.

**TABLE 4:** Performances of all algorithms in the subgroups based on WMH load (10 subjects with lowest vs. 10 subjects with highest) compared to the reference segmentation at volume- and lesion- level.

Method	Subgroup	Whole brain volumes			Lesions		
		**DSC**	**Sensitivity**	**Precision**	**F1-score**	**Sensitivity**	**Precision**
BIANCA	Low	0.582 ± 0.123	0.668 ± 0.170	0.532 ± 0.126	0.522 ± 0.137	0.590 ± 0.166	0.493 ± 0.157
	High	0.752 ± 0.096	0.668 ± 0.157	0.894 ± 0.060	0.593 ± 0.103	0.575 ± 0.125	0.642 ± 0.132
LST LGA	Low	0.444 ± 0.192	0.416 ± 0.249	0.563 ± 0.098	0.482 ± 0.120	0.379 ± 0.120	0.691 ± 0.134
	High	0.690 ± 0.089	0.637 ± 0.146	0.785 ± 0.097	0.415 ± 0.105	0.348 ± 0.115	0.585 ± 0.195
LST LPA FLAIR only	Low	0.610 ± 0.111	0.631 ± 0.234	0.674 ± 0.148	0.569 ± 0.144	0.528 ± 0.205	0.703 ± 0.206
	High	0.708 ± 0.125	0.763 ± 0.183	0.716 ± 0.183	0.469 ± 0.143	0.454 ± 0.145	0.563 ± 0.216
LST LPA T1w + FLAIR	Low	0.524 ± 0.154	0.518 ± 0.267	0.660 ± 0.112	0.590 ± 0.184	0.500 ± 0.237	0.839 ± 0.137
	High	0.680 ± 0.116	0.721 ± 0.207	0.705 ± 0.157	0.454 ± 0.162	0.408 ± 0.146	0.582 ± 0.218
pgs	Low	0.482 ± 0.202	0.432 ± 0.211	0.615 ± 0.158	0.502 ± 0.144	0.501 ± 0.203	0.557 ± 0.169
	High	0.773 ± 0.133	0.717 ± 0.180	0.883 ± 0.059	0.713 ± 0.159	0.680 ± 0.155	0.751 ± 0.166
sysu_media (default)	Low	0.442 ± 0.202	0.438 ± 0.210	0.457 ± 0.199	0.439 ± 0.150	0.537 ± 0.274	0.395 ± 0.143
	High	0.766 ± 0.107	0.743 ± 0.164	0.826 ± 0.075	0.633 ± 0.119	0.654 ± 0.128	0.623 ± 0.133
sysu_media (retrained)	Low	0.608 ± 0.120	0.623 ± 0.212	0.648 ± 0.085	0.635 ± 0.125	0.573 ± 0.194	0.759 ± 0.103
	High	0.753 ± 0.081	0.712 ± 0.147	0.829 ± 0.090	0.637 ± 0.099	0.591 ± 0.111	0.727 ± 0.165

Mean and standard deviation are displayed. DSC, Sørensen-Dice similarity coefficient; T1w, T1-weighted image; FLAIR, fluid-attenuated inversion recovery image; BIANCA, brain intensity abnormality classification algorithm; LST LGA/-LPA, lesion segmentation toolbox for SPM using lesion growth algorithm/lesion prediction algorithms.

## Discussion

The objective of the present study was to give an overview of the performances of automatic WMH segmentation tools in the memory clinic context, notably by comparing more traditional tools and new deep learning algorithms in a multicenter sample of cognitive impairment and dementia [DELCODE study (16)]. Our results showed that the new class of algorithms based on a deep learning approach (pgs, sysu_media) performed only marginally better or in the same range as some of the more established tools (LST LPA, FSL BIANCA). Best performances and lowest variability were generally achieved for sysu_media, retrained on the DELCODE subset, compared to other tools considered in this evaluation. Even if the overall performances were quite moderate (best DSC is around 0.7), all methods showed an excellent volume consistency compared with the RS. After discussing advantages and challenges of the evaluated tools, we provide recommendations for future studies including automated WMH detection in a multicenter context.

A comparison with the past literature on the evaluation of automatic WMH segmentation tools is complex due to multiple factors, including differences in algorithms being evaluated, disparate data (in terms of quantity and quality) used for evaluation, parameters of the tools tuned or not, and heterogeneous evaluation measures being reported (27). In this study, we thus computed performance measures already implemented in previous studies (6, 11, 12) to enhance comparison across studies and to get simple but efficient performance indices widely used in clinical and research practice. Thus, it is to note first that the initial performance estimations of tools are often evaluated in a single center, with tuned parameters leading to better performances, and thus cannot be generalized to other datasets. For example, the high performances reported in the original paper of LST LGA (7) were not replicated in a series of comparative studies (6, 11). Of note, LST LGA was developed in the context of multiple sclerosis, not for WMH of presumed vascular origin. Even if WMH are visually similar, underlying pathologies are not the same and anatomical location may differ, especially for small lesions.

In general, our results are consistent with previous studies using similar approaches. Indeed, Heinen et al. (11) observed best performances for kNN-TMP and to a lesser extent for LST LPA (with FLAIR only) over other methods such as LST LGA. Unfortunately, no algorithm using deep learning was evaluated in this paper. Another previous study (6) compared traditional and deep learning algorithms and obtained best performances for the deep learning methods [NicMSlesion (28)] and the LST LPA, depending on the dataset that was assessed (resp. research or clinical setting). Interestingly, for the deep learning algorithm, the authors observed significantly better performances when retraining was performed using a subset of the evaluated dataset, compared with the default training dataset. We observed the same with our evaluation. This observation is not surprising, but it highlights the importance of the retraining approach (even with a small sample, 20 images here) on study datasets to achieve optimized segmentation results for deep learning algorithms. Moreover, Vanderbecq et al. (6) showed that with the deep learning tool, the variability in the WMH segmentations among participants could be large (for the research dataset) or narrow (for the clinical dataset). In our comparison, the variability of our performance measures was the smallest for sysu_media, retrained on the DELCODE subset. This observation highlights the capacity of deep learning algorithms to provide more consistent results. This narrow variability is especially important for multicenter cohorts, where acquisition and reconstruction parameters may vary between centers, notably in terms of scanner manufacturer, field strength, signal-to-noise ratio, sequences parameters or even pathology levels. However, more studies are needed to increase our knowledge about the effect of these parameters on the accuracy of automated WMH segmentations.

In addition, segmentation performances were evaluated using the total number of lesions and their location. These two measures of interest as derived from the STRIVE criteria (Table 3) (1) often not reported in comparisons of automated WMH segmentation tools. Here, we used a more refined method for the detection of lesions present in the manual RS (5), to go beyond mere counting of the number of lesions (29). As expected from our results for global WMH volumes, the performances across the different tools for the detection of the total number of lesions were moderate. Overall, sysu_media retrained on the DELCODE subset performed the best, closely followed by pgs. In both cases, a high precision was coupled with lower sensitivity, a behavior that was found for all algorithms but sysu_media (trained on the MICCAI dataset). This pattern reflects the capacity of these algorithms to detect fewer lesions than expected, resulting in an under-estimation of the lesions. Overall, both precision and sensitivity have to be evaluated in line with all parameters to have an impression of the overall performance of each algorithm. Second, we also found that the location of the lesion is important, especially because of the underlying nature of the tissue. For example, a study reported different magnetization transfer ratio in frontal than in occipital WMH, which may reflect either a different stage of tissue damage or possibly different underlying tissue damage (30). Our results essentially demonstrate that all parts of the brain are not segmented with the same quality and the performances varied with the tools used. These results highlight that it seems to be difficult for most of the tools to segment all regions with a consistent high accuracy. This issue may be related to the presence of image distortions due to bias fields inherent to MR imaging, since most of the WMH segmentation tools rely on voxel intensity.

Furthermore, we studied the impact of high vs. low WMH burden on the quality of automated WMH segmentations, by splitting our test subset (*n* = 30) *post hoc* into two subgroups. Our results confirm previous observations reporting better performances for the higher compared to lower WMH load for all tools (6, 11, 12) at the global volume level. This result is most probably positively biased by performance measures, which give more weight to large compared to small lesions. Interestingly, similar to an earlier study (12), we observed an improvement with the deep learning tool sysu_media (retrained on the DELCODE subset) for the detection of small lesions, while the results are more disparate for the other tools. These good performances for the retrained sysu_media for lesions of different sizes may be due to the fact that deep learning methods are capable of incorporating a high degree of feature combinations, such as the location or shape of the lesions than voxel intensities solely as for non-deep learning.

Finally, we want to provide recommendations for future studies on WMH and their segmentation by automatic tools. First, studies should include a clear definition of WMH. Accordingly to Frey et al. (15), only 18% of articles on WMH reported an explicit definition. The situation is getting slightly better since the introduction of the consensus criteria from STRIVE (1). The lack of clear reference leads to a real difficulty in understanding the processes underlying WMH and makes it almost impossible to reproduce previous results. Second, the image quality and data preprocessing procedures are central points to get accurate measures of WMH. It is true for mono-centric studies, but even more for multicenter studies since different MR systems and sequence parameters are commonly used. A standardized or at least harmonized protocol should thus be implemented in any multi-center study to minimize the variability of image quality. After image acquisition, some preprocessing may be applied to optimize subsequent WMH segmentation. However, pre-processing might also introduce negative effects that one needs to be aware of, such as fading away of the border of the lesions due to too strong bias correction [see Figure 10 of Wardlaw et al. (31) for a compelling example]. Our results also suggested better segmentation results for LST LPA with FLAIR only as input compared to the same algorithm with T1w + FLAIR images, potentially suggesting a loss of information through registration due to repeated interpolation and/or rounding of image intensity values. Third, other methodological considerations may help to increase the accuracy of automated segmentations. One interesting point to consider could be the addition of an individual WM mask, which could improve the segmentation by masking out non-WM areas. This mask should be checked carefully, since traditional brain compartment segmentation tools may consider WMH as belonging to the nearly similar voxel intensity of GM and thus exclude them from a WM mask (32). However, recently some efforts were made by the developers of neuroimaging software to classify WMH into a distinct tissue class, for instance, for FreeSurfer (33).

Overall, our results suggest that the choice of the automated WMH segmentation tool is important, including the advantages and drawbacks of each specific tool for the desired context. As we have shown in our study, some tools may provide more reliable results for specific brain regions or may be able to better detect small lesions, while others may not. When applying such tools, we further recommend to explicitly state the specific name and version used, since the field of WMH segmentation is in constant evolution and so are the related tools. The initial parameters of the tools (such as the kappa threshold for LST LGA) but also the threshold of the output probability maps should also be tuned according to the studied dataset. Indeed, default values of tools should be adapted to better fit own specifications that may vary from the original publication. Finally, the model parameters should be optimized by retraining when using deep learning algorithms to improve the WMH segmentation results as shown above.

A few limitations to our study need to be reported. First, we used only one trained rater for the RS. Some studies have shown that the inter-rater reliability may be low for WMH detection (34). However, this problem was mostly found in studies published before the consensus definitions of WMH as described by the STRIVE criteria (1). Indeed, more recent studies tend to have a reduced variability between and/or within raters [inter/intra-rater ICC > 0.97 (6, 11)]. This led us to the decision that one trained rater with quality assurance using a step-by-step manual segmentation protocol and continuous monitoring by experts would be sufficient in our study. Second, we acknowledge that the number of participants in our dataset may be low. The limitation here was notably due to the time required to segment each FLAIR image manually, which was more than 8 h for those participants with heavy WMH burden. We thus wanted to focus more on a qualitative dataset, extremely carefully segmented and documented, than on a quantitative dataset realized quickly at the expense of good quality. At the end, using only 2 participants per DELCODE center for the training subset, we show that the deep learning method sysu_media performed best and with the lowest variability for most of the measures. Hence, the size of a given training dataset may not be such an issue compared to the importance of giving the algorithm a chance to adapt to the properties of the sample and desired segmentation performance. Nevertheless, the quality of segmentation per scanner could not be evaluated with such small groups. Furthermore, all images were solely acquired on Siemens scanners using a harmonized protocol. Thus, we acknowledge that our results may not be replicable with other machines and differing protocols between images. Of note, a previous study showed that performances of LST-LGA and Bianca (and LST-LPA to a lesser extent) algorithms were particularly robust to different scanners (6). We also decided not to apply final masking to exclude regions excluded in the manual segmentation (see “Reference segmentation procedure” section). We acknowledge that this decision might unfairly bias the results in favor of methods with a retrained dataset. Finally, the choice of measures for the evaluation of performances is always a matter of debate. Some efforts have been made to propose guidelines with metrics suitable for the evaluation of segmentation (35). But in practice, it is always difficult to use multiple results in a single paper without overwhelming readers with too much information, especially when the evaluation is not only globally, but at multiple levels, as in our study. We thus favored to use simple reliable metrics also used in many other comparative studies and applied these metrics not only at the whole brain voxel level, but also in relation to the total number of WMH lesions and their location.

To summarize, we showed that deep learning algorithms, especially when retrained, performed the best in the present multicenter context of cognitive impairment and AD dementia. Nevertheless, their performances remain close to traditional methods.

## Data availability statement

The data that support findings of the present study are available on reasonable request from the DELCODE database. Requests to access these datasets should be directed to the German Center for Neurodegenerative Diseases [Deutsches Zentrum für Neurodegenerative Erkrankungen e.V. (DZNE)], Bonn. Respective scripts for data analysis are available from the corresponding author (MG) on reasonable request. The adapted sysu_media implementation and DELCODE model weights are available as an open-source package on GitHub (https://github.com/0rC0/WMHpypes).

## Ethics statement

The general study protocol for the DELCODE study was approved by the ethical committees of the medical faculties of all sites, i.e., the Ethical Committees of Berlin (Charité – Universitätsmedizin), Bonn (Medical Faculty, University of Bonn), Cologne (Medical Faculty, University of Cologne), Göttingen (Universitätsmedizin Göttingen), Magdeburg (Medical Faculty, Otto-von-Guericke University, Magdeburg), Munich (Medical Faculty, Ludwig-Maximilians-Universität), Rostock (Medical Faculty, University of Rostock), and Tübingen (Medical Faculty, University of Tübingen). The process was led and coordinated by the ethical committee of the medical faculty of the University of Bonn under the registration number: 171/13. The patients/participants provided written informed consent to participate in the DELCODE study.

## DELCODE study group

Amthauer Holger, Cetindag Arda Can, Cosma Nicoleta Carmen, Diesing Dominik, Ehrlich Marie, Fenski Frederike, Freiesleben Silka Dawn, Fuentes Manuel, Hauser Dietmar, Hujer Nicole, Incesoy Enise Irem, Kainz Christian, Lange Catharina, Lindner Katja, Megges Herlind, Peters Oliver, Preis Lukas, Altenstein Slawek, Lohse Andrea, Franke Christiana, Priller Josef, Spruth Eike, Villar Munoz Irene, Barkhoff Miriam, Boecker Henning, Brosseron Frederic, Daamen Marcel, Engels Tanja, Faber Jennifer, Fließbach Klaus, Frommann Ingo, Grobe-Einsler Marcus, Hennes Guido, Herrmann Gabi, Jost Lorraine, Kalbhen Pascal, Kimmich Okka, Kobeleva Xenia, Kofler Barbara, McCormick Cornelia, Miebach Lisa, Miklitz Carolin, Müller Anna, Oender Demet, Polcher Alexandra, Purrer Veronika, Röske Sandra, Schneider Christine, Schneider Anja, Spottke Annika, Vogt Ina, Wagner Michael, wolfsgruber Steffen, Yilmaz Sagik, Bartels Claudia, Dechent Peter, Hansen Niels, Hassoun Lina, Hirschel Sina, Nuhn Sabine, Pfahlert Ilona, Rausch Lena, Schott Björn, Timäus Charles, Werner Christine, Wiltfang Jens, Zabel Lioba, Zech Heike, Bader Abdelmajid, Baldermann Juan Carlos, Dölle Britta, Drzezga Alexander, Escher Claus, Ghiasi Nasim Roshan, Hardenacke Katja, Jessen Frank, Lützerath Hannah, Maier Franziska, Marquardt Benjamin, Martikke Anja, Meiberth Dix, Petzler Snjezana, Rostamzadeh Ayda, Sannemann Lena, Schild Ann-Katrin, Sorgalla Susanne, Stockter Simone, Thelen Manuela, Tscheuschler Maike, Uhle Franziska, Zeyen Philip, Bittner Daniel, Cardenas-Blanco Arturo, Dobisch Laura, Düzel Emrah, Grieger-Klose Doreen, Hartmann Deike, Metzger Coraline, Nestor Peter, Ruß Christin, Schulze Franziska, Speck Oliver, Wenzel Glanz, Yakupov Renat, Ziegler Gabriel, Brauneis Christine, Bürger Katharina, Catak Cihan, Coloma Andrews Lisa, Dichgans Martin, Dörr Angelika, Ertl-Wagner Birgit, Frimmer Daniela, Huber Brigitte, Janowitz Daniel, Kreuzer Max, Markov Eva, Müller Claudia, Rominger Axel, Schmid (ehemals Spreider) Jennifer, Seegerer Anna, Stephan Julia, Zollver Adelgunde, Burow Lena, de Jonge Sylvia, Falkai Peter, Garcia Angarita Natalie, Görlitz Thomas, Gürsel Selim Üstün, Horvath Ildiko, Kurz Carolin, Meisenzahl-Lechner Eva, Perneczky Robert, Utecht Julia, Dyrba Martin, Janecek-Meyer Heike, Kilimann Ingo, Lappe Chris, Lau Esther, Pfaff Henrike, Raum Heike, Sabik Petr, Schmidt Monika, Schulz Heike, Schwarzenboeck Sarah, Teipel Stefan, Weber Marc-Andre, Buchmann Martina, Heger Tanja, Hinderer Petra, Kuder-Buletta Elke, Laske Christoph, Munk Matthias, Mychajliw Christian, Soekadar Surjo, sulzer Patricia, and Trunk Theresia.

## Author contributions

OP, LP, JP, ES, ASc, KF, JW, BS, FM, WG, KB, DJ, RP, B-SR, ST, IK, CLas, MM, ASp, NR, LD, ME, PD, JH, KS, ED, and FJ: conception of the DELCODE study and acquisition of the data. MG, AD’O, CLan, IZ, and MW: analyses of the present data. MG, AD’O, CLan, AG-C, MDy, MDu, GZ, and MW: interpretation of the present data and manuscript drafting and/or revision. All authors contributed to the article and approved the final version.

## Footnotes

1
itksnap.org

2
https://www.fil.ion.ucl.ac.uk/spm/

3
https://wmh.isi.uu.nl/results/

4
http://www.neuro.uni-jena.de/cat/

5
https://github.com/0rC0/WMHpypes
