# Supplementary material for: Performance evaluation of automated white matter hyperintensity segmentation algorithms in a multicenter cohort on cognitive impairment and dementia
Source: Front Psychiatry. 2023 Jan 12;13:1010273. doi: 10.3389/fpsyt.2022.1010273 (PMC9877422; doi:10.3389/fpsyt.2022.1010273)
Supplement: Supplementary file 1 [file Data_Sheet_1.PDF]

Supplementary information

Performance evaluation of automated white matter hyperintensity segmentation algorithms in a multicenter cohort on cognitive impairment and dementia

Malo Gaubert[1*], Andrea Dell'Orco[1, 2], Catharina Lange[1, 3], Antoine Garnier-Crussard[4, 5, 6], Isabella Zimmermann[1], Martin Dyrba[7], Marco Duering[8], Gabriel Ziegler[9], Oliver Peters[10, 11], Lukas Preis[11], Josef Priller[10, 12, 13, 14], Eike Jakob Spruth[10, 12], Anja Schneider[15, 16], Klaus Fliessbach[15, 16], Jens Wiltfang[17, 18, 19], Björn H.Schott[17, 18], Franziska Maier[20], Wenzel Glanz[9], Katharina Buerger[21, 22], Daniel Janowitz[22], Robert Perneczky[21, 22, 23, 24, 25], Boris-Stephan Rauchmann[22], Stefan Teipel[7, 26], Ingo Kilimann[7, 26], Christoph Laske[27, 28], Matthias H. Munk[27, 28], Annika Spottke[15, 29], Nina Roy[15], Laura Dobisch[9], Michael Ewers[21], Peter Dechent[30], John Dylan Haynes[31], Klaus Scheffler[32], Emrah Düzel[9, 33], Frank Jessen[15, 20, 34], Miranka Wirth[1*] for the DELCODE study group

Corresponding authors (*):

MSc Malo Gaubert (malo.gaubert@dzne.de)

Dr. Miranka Wirth (miranka.wirth@dzne.de)

Table of contents

Computational burden 3
Definition of the regions of interest (ROI) and processing........ 4
DSC for optimal parameters of each tool on training sub-dataset 6
Evolution of DSC according to the evolution of the thresholding of probability maps for LST LPA (FLAIR only and T1w+FLAIR), pgs, sysu_media (default and retrained)........ 7
Evolution of DSC according to the evolution of the thresholding of probability maps and of the WM mask thresholding for BIANCA 8
Evolution of DSC according to the evolution of the thresholding of probability maps and of the kappa index for LST LGA 9
Performance of all algorithms in regions of interest 10
Example of poor WMH segmentation for all algorithms 12
References........ 13

Computational burden

All processing (training or testing) were performed on a workstation including 28 double core processors Intel(R) Xeon(R) Gold 6132 CPU @ 2.60GHz 64bits, 256 Gb of DDR4 RAM. A graphical processor unit (GPU) Nvidia Quadro P5000 was also used for sysu_media retraining on DELCODE sub-dataset. The workstation is running on Ubuntu 20.04.3 LTS. The processing time for one subject were the following:

Tool	Preprocessing not included in the tool (per subject)	Training	Testing
**BIANCA**	WM mask creation: ~29min<br>BET: ~5min	50s	11s
**LST LGA**	Included in toolbox	NA	~6min30
**LST LPA with FLAIR only**	Included in toolbox	NA	~3min
**LST LPA with T1w+FLAIR**	Included in toolbox	NA	~3min30
**pgs**	Coregistration: 30s	NA	~6min
**sysu_media (default)**	Included in toolbox	NA	~2min
**sysu_media (retrained)**	BET: ~5min<br>SANLM Denoising: ~1min	~3h	~2min

Legend: WM=white matter; BET=brain extraction tool; T1w=T1-weighted image; FLAIR= Fluid-attenuated inversion recovery image; BIANCA=brain intensity abnormality classification algorithm; LST LGA/-LPA=Lesion segmentation toolbox for SPM using lesion growth algorithm/ lesion prediction algorithm; min=minute; s=second; NA=not applicable.

Definition of the regions of interest (ROI) and processing

Six regions of interest have been derived from Hammersmith atlas [1]. The following regions have been merged using SPM module “imcalc” to define the ROI:

- **Corpus callosum**: left Corpus Callosum, right Corpus Callosum;
- **Frontal cortex**: Left Anterior Cinguli Gyrus, Right Anterior Cinguli Gyrus, Left Middle Frontal Gyrus, Right Middle Frontal Gyrus, Left Precentral Gyrus, Right Precentral Gyrus, Left Gyrus Rectus, Right Gyrus Rectus, Left Orbito-Frontal Gyri, Right Orbito-Frontal Gyri, Left Inferior Frontal Gyrus, Right Inferior Frontal Gyrus, Left Superior Frontal Gyrus, Right Superior Frontal Gyrus;
- **Insular cortex**: Left Insula, Right Insula;
- **Occipital cortex**: Left Lateral Occipital Lobe, Right Lateral Occipital Lobe, Left Lingual Gyrus, Right Lingual Gyrus, Left Cuneus, Right Cuneus
- **Parietal cortex**: Left Posterior Cinguli Gyrus, Right Posterior Cinguli Gyrus, Left Inferior Lateral Pariatal Lobe, Right Inferior Lateral Pariatal Lobe, Left Postcentral Gyrus, Right Postcentral Gyrus, Left Superior Parietal Gyrus, Right Superior Parietal Gyrus
- **Temporal cortex**: Left Hippocampus, Right Hippocampus, Left Amygdala, Right Amygdala, Left Anterior Medial Temporal Lobe, Right Anterior Medial Temporal Lobe, Left Anterior Lateral Temporal Lobe, Right Anterior Lateral Temporal Lobe, Left Ambient and Parahippocampus Gyri, Right Ambient and Parahippocampus Gyri, Left Superior Temporal Gyrus, Right Superior Temporal Gyrus, Left Inferior Middle Temporal Gyri, Right Inferior Middle Temporal Gyri, Left Fusiform Gyrus, Right Fusiform Gyrus, Left Posterior Temporal Lobe, Right Posterior Temporal Lobe

Individual T1w images were segmented and normalized to MNI space using the computational anatomy toolbox (CAT12; v6, release 1450 [2]) for SPM12 in order to get deformation fields from MNI space to individual T1w spaces. Then, the deformation fields were applied to send back the ROI into T1w spaces using nearest neighbor as interpolation method. Finally, the module “coregister estimate

& reslice” (nearest neighbor as interpolation method) with FLAIR images as references and T1w images as images to move were used to register the warped ROI into FLAIR space.

DSC for optimal parameters of each tool on training sub-dataset

Tool	Parameters	Probability map thresholding	DSC
**BIANCA**	WM mask thresholding: 0.3	0.60	0.661
**LST LGA**	Kappa = 0.1	0.35	0.564
**LST LPA FLAIR only**	NA	0.10	0.676
**LST LPA T1w+FLAIR**	NA	0.15	0.606
**pgs**	NA	0.15	0.621
**sysu_media (default)**	NA	0.65	0.594
**sysu_media (retrained)**	NA	0.45	0.708

Average DSC reported with optimal parameters for each tested tool on images from training sub-dataset. Legend: WM: white matter; DSC= Sørensen-Dice similarity coefficient; T1w=T1-weighted image; FLAIR= Fluid-attenuated inversion recovery image; BIANCA=brain intensity abnormality classification algorithm; LST LGA/-LPA=Lesion segmentation toolbox for SPM using lesion growth algorithm/ lesion prediction algorithm; NA=not applicable.

Evolution of DSC according to the evolution of the thresholding of probability maps for LST LPA (FLAIR only and T1w+FLAIR), pgs, sysu_media (default and retrained)

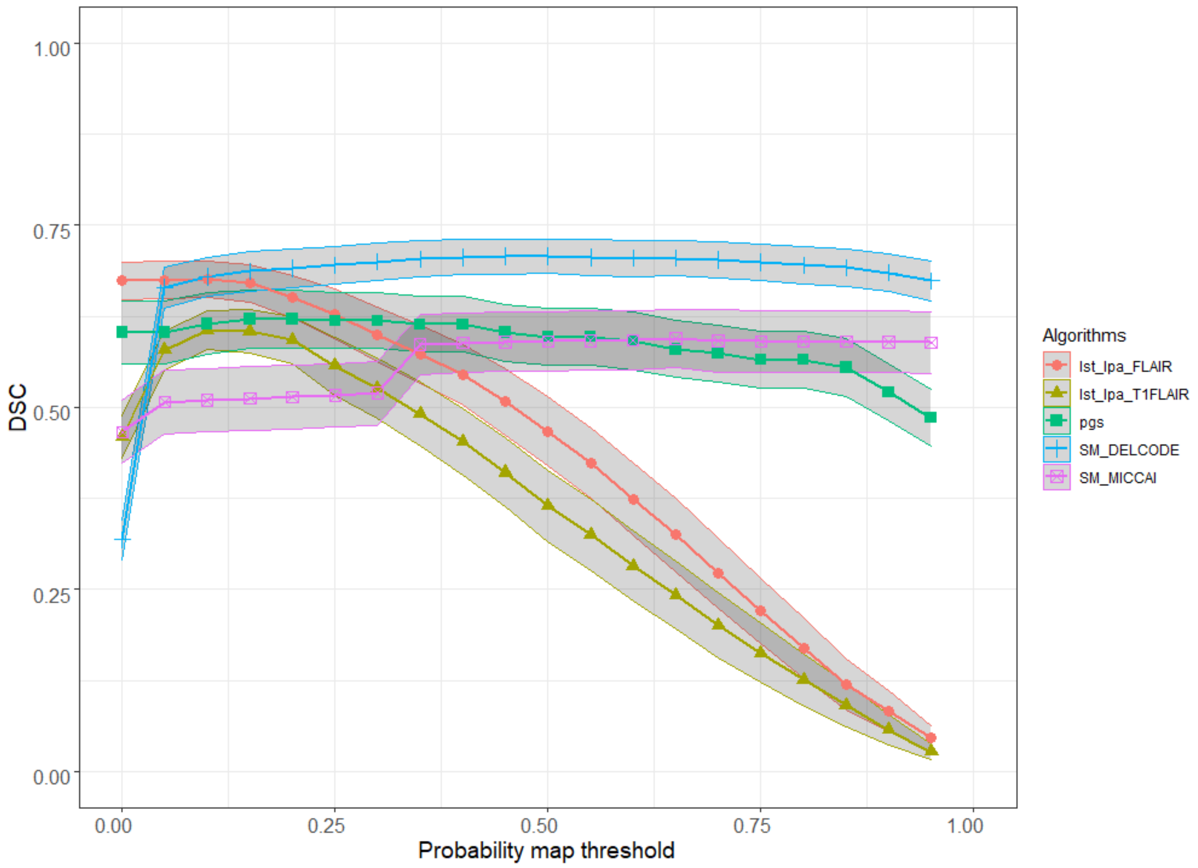

Average DSC (and standard deviation in light gray) for LST LPA with FLAIR only (red), LST LPA with T1w and FLAIR (olive), pgs (green), sysu media retrained on DELCODE training sub-dataset (retrained, blue) and sysu media trained on MICCAI dataset (default, purple) according to probability map threshold. Legend: DSC= Sørensen-Dice similarity coefficient; T1w=T1-weighted image; FLAIR= Fluid-attenuated inversion recovery image; LST LGA/-LPA=Lesion segmentation toolbox for SPM using lesion growth algorithm/ lesion prediction algorithm.

Evolution of DSC according to the evolution of the thresholding of probability maps and of the WM mask thresholding for BIANCA

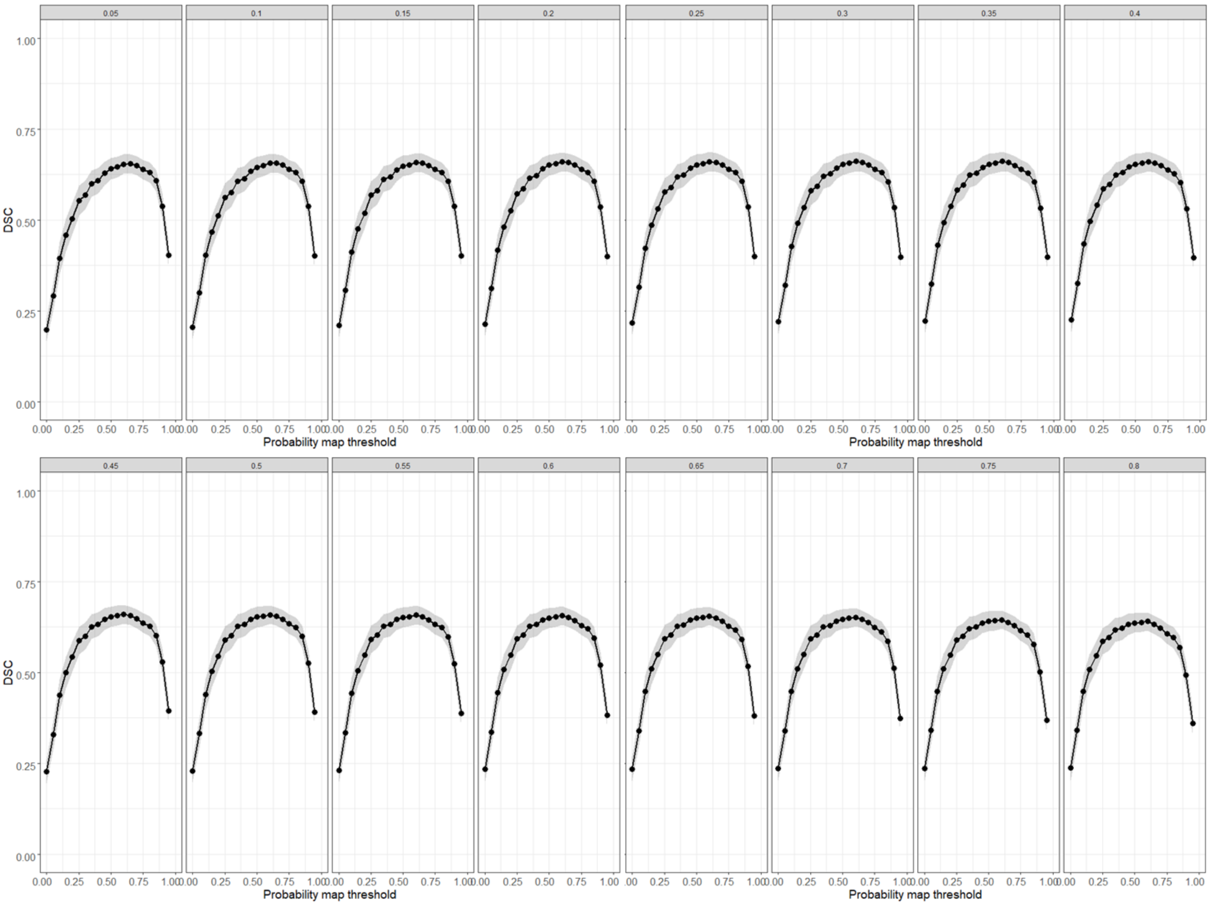

Each graph represents the average DSC (and standard deviation in light gray) according to probability map threshold for different values of white matter mask threshold (top gray header of each graph) used by BIANCA. Legend: DSC= Sørensen-Dice similarity coefficient; BIANCA=brain intensity abnormality classification algorithm.

Evolution of DSC according to the evolution of the thresholding of probability maps and of the kappa index for LST LGA

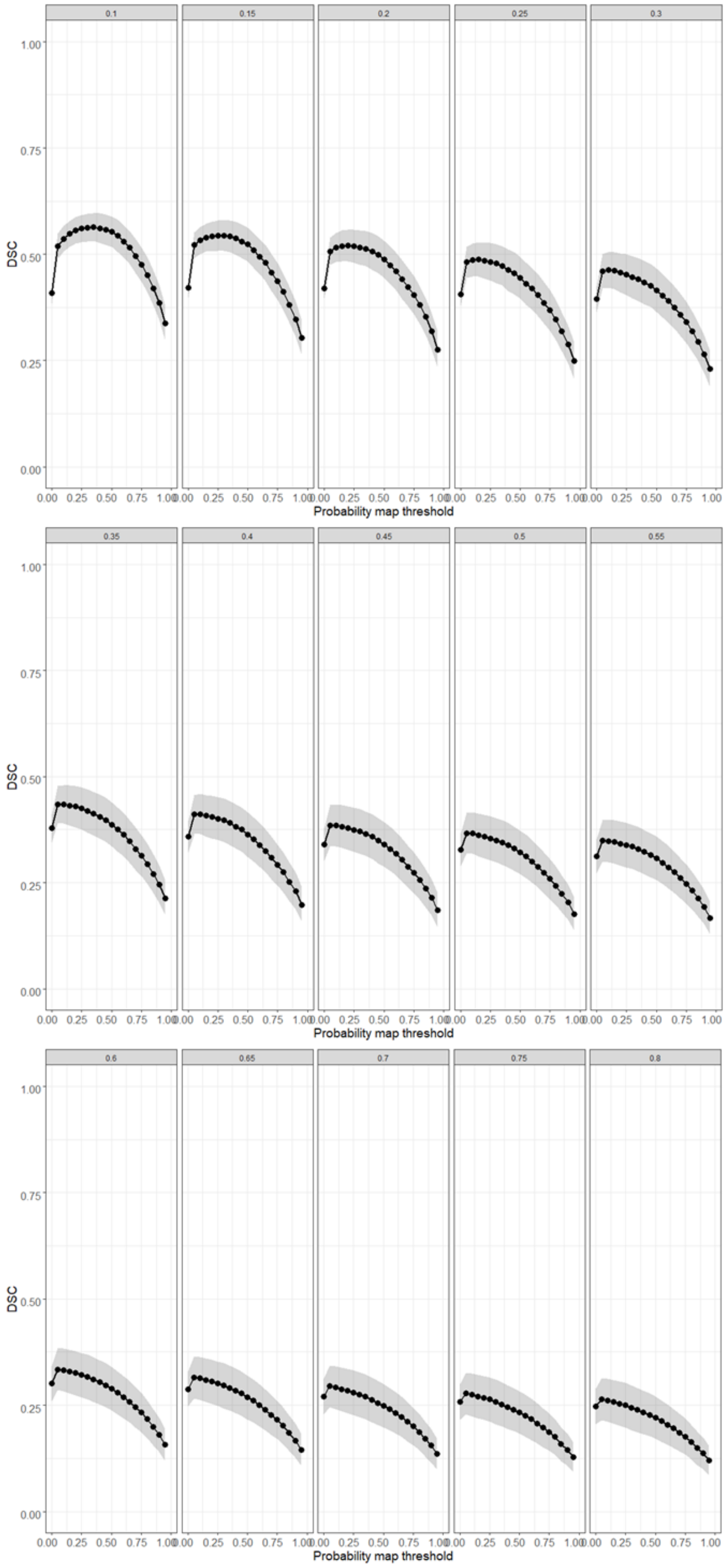

Each graph represents the average DSC (and standard deviation in light gray) according to probability map threshold for different values of kappa (top gray header of each graph) used by LST LGA. Legend: DSC= Sørensen-Dice similarity coefficient; LST LGA =Lesion segmentation toolbox for SPM using lesion growth algorithm.

Performance of all algorithms in regions of interest

ROI	Method	Global WMH volume (ml)	DSC	Sensitivity	Precision
Corpus Callosum	Reference	.84 ± .57	NA	NA	NA
	BIANCA	.68 ± .39	.709 ± .125	.643 ± .174	.836 ± .098
	LST LGA	.91 ± .66	.680 ± .159	.679 ± .218	.734 ± .098
	LST LPA FLAIR only	.94 ± .65	.773 ± .083	.813 ± .166	.777 ± .123
	LST LPA T1w+FLAIR	.97 ± .70	.704 ± .120	.751 ± .208	.715 ± .110
	pgs	.49 ± .39	.467 ± .199	.351 ± .185	.858 ± .117
	sysu_media default	.53 ± .42	.448 ± .200	.346 ± .186	.762 ± .183
	sysu_media retrained	.83 ± .46	.769 ± .073	.780 ± .124	.780 ± .098
Frontal	Reference	2.55 ± 3.34	NA	NA	NA
	BIANCA	1.90 ± 2.14	.722 ± .143	.700 ± .187	.783 ± .169
	LST LGA	2.29 ± 3.19	.617 ± .166	.637 ± .219	.642 ± .186
	LST LPA FLAIR only	2.38 ± 3.30	.681 ± .157	.702 ± .242	.756 ± .164
	LST LPA T1w+FLAIR	2.34 ± 3.28	.625 ± .201	.644 ± .274	.716 ± .147
	pgs	2.64 ± 3.32	.754 ± .145	.825 ± .157	.722 ± .176
	sysu_media default	3.07 ± 3.74	.672 ± .207	.832 ± .154	.595 ± .229
	sysu_media retrained	2.16 ± 2.58	.698 ± .140	.699 ± .199	.752 ± .150
Insula	Reference	.20 ± .28	NA	NA	NA
	BIANCA	.18 ± .21	.518 ± .271	.646 ± .277	.554 ± .321
	LST LGA	.19 ± .29	.510 ± .266	.526 ± .315	.647 ± .222
	LST LPA FLAIR only	.24 ± .37	.572 ± .265	.672 ± .290	.660 ± .276
	LST LPA T1w+FLAIR	.24 ± .41	.489 ± .281	.581 ± .325	.599 ± .263
	pgs	.18 ± .29	.688 ± .265	.702 ± .263	.797 ± .218
	sysu_media default	.20 ± .30	.673 ± .272	.699 ± .288	.720 ± .281
	sysu_media retrained	.17 ± .23	.538 ± .260	.575 ± .289	.640 ± .264
Occipital	Reference	.91 ± 1.21	NA	NA	NA
	BIANCA	.67 ± .78	.717 ± .186	.656 ± .222	.876 ± .096
	LST LGA	.73 ± .95	.539 ± .282	.502 ± .309	.776 ± .128
	LST LPA FLAIR only	.89 ± 1.25	.660 ± .222	.634 ± .291	.837 ± .137
	LST LPA T1w+FLAIR	.81 ± 1.13	.552 ± .279	.516 ± .330	.836 ± .135
	pgs	.78 ± .93	.706 ± .196	.688 ± .242	.806 ± .145
	sysu_media default	.75 ± .88	.693 ± .211	.672 ± .255	.795 ± .123
	sysu_media retrained	.86 ± .91	.674 ± .173	.717 ± .227	.705 ± .155
Parietal	Reference	2.05 ± 3.59	NA	NA	NA
	BIANCA	1.40 ± 2.02	.690 ± .179	.680 ± .221	.781 ± .171
	LST LGA	1.31 ± 2.45	.478 ± .221	.395 ± .225	.752 ± .199
	LST LPA FLAIR only	1.92 ± 3.02	.641 ± .183	.688 ± .267	.706 ± .169
	LST LPA T1w+FLAIR	1.74 ± 2.71	.589 ± .189	.601 ± .276	.707 ± .162
	pgs	1.81 ± 3.00	.671 ± .198	.688 ± .243	.710 ± .193
	sysu_media default	1.99 ± 3.27	.647 ± .214	.716 ± .236	.636 ± .236
	sysu_media retrained	1.64 ± 2.43	.656 ± .169	.661 ± .206	.693 ± .203
Temporal	Reference	.97 ± 1.87	NA	NA	NA
	BIANCA	.65 ± 1.11	.619 ± .203	.604 ± .201	.713 ± .243
	LST LGA	.65 ± 1.33	.466 ± .253	.418 ± .238	.630 ± .262

LST LPA FLAIR only	1.08 ± 1.72	.627 ± .208	.686 ± .253	.669 ± .208
LST LPA T1w+FLAIR	.94 ± 1.53	.574 ± .225	.589 ± .266	.681 ± .200
pgs	.65 ± 1.33	.616 ± .257	.551 ± .238	.861 ± .176
sysu_media default	.73 ± 1.42	.642 ± .226	.587 ± .235	.804 ± .139
sysu_media retrained	.70 ± 1.18	.638 ± .181	.661 ± .224	.681 ± .165

Average WMH volume, DSC, sensitivity and precision for all algorithms in the 6 ROI. Legend: ROI=region of interest; ml=milliliter; DSC= Sørensen-Dice similarity coefficient; ICC=intra-class correlation; T1w=T1-weighted image; FLAIR=fluid-attenuated inversion recovery image; BIANCA=brain intensity abnormality classification algorithm; LST LGA/-LPA=lesion segmentation toolbox for SPM using lesion growth algorithm/lesion prediction algorithm; NA=not applicable.

Example of poor WMH segmentation for all algorithms

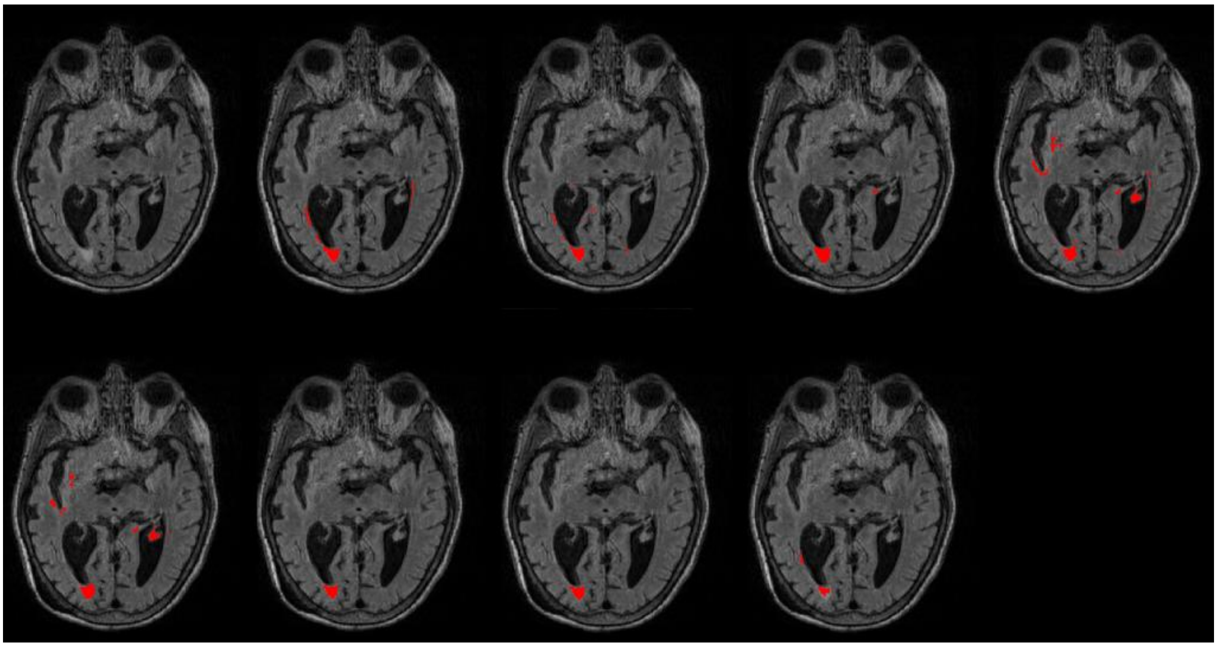

Example of the WMH segmentation for one subject with poor segmentation performances. (top line, from left to right): original image, manual segmentation, BIANCA, LST LGA, LST LPA FLAIR only; (bottom line, from left to right): LST LPA T1w+FLAIR, PGS, sysu_media (default), sysu_media (retrained).

References

1. Hammers, A. *et al.* Three-dimensional maximum probability atlas of the human brain, with particular reference to the temporal lobe. *Hum. Brain Mapp.* **19**, 224–247 (2003).

2. Gaser, C. et al. A Computational Anatomy Toolbox for the Analysis of Structural MRI Data. Neuroimage, in review.
